# Prenatal care of Venezuelans in Colombia: migrants navigating the healthcare system

**DOI:** 10.11606/s1518-8787.2021055002999

**Published:** 2021-08-06

**Authors:** Vanesa Giraldo, Rita Sobczyk, Julián Alfredo Fernández-Niño, Maylen Liseth Rojas-Botero, Ietza Bojorquez

**Affiliations:** I University of Massachusetts Department of Anthropology AmherstMA USA University of Massachusetts. Department of Anthropology. Amherst, MA, USA; II Universidad del Norte Departamento de Historia y Ciencias Sociales BarranquillaATL Colombia Universidad del Norte. Departamento de Historia y Ciencias Sociales. Barranquilla, ATL, Colombia; III Universidad del Norte Departamento de Salud Pública BarranquillaATL Colombia Universidad del Norte. Departamento de Salud Pública. Barranquilla, ATL, Colombia; IV Universidad de Antioquia Programa de Doctorado en Salud Pública MedellínANT Colombia Universidad de Antioquia. Programa de Doctorado en Salud Pública. Medellín, ANT, Colombia; V El Colegio de la Frontera Norte Departamento de Estudios de Población TijuanaBC México El Colegio de la Frontera Norte. Departamento de Estudios de Población. Tijuana, BC, México

**Keywords:** Pregnant Women, Prenatal Care, Health Services Accessibility, Social Capital, Transients and Migrants

## Abstract

**OBJECTIVES:**

To explore the experiences of irregular (undocumented) Venezuelan migrants in accessing prenatal health services in Colombia and to examine the economic, social, and cultural resources mobilized by them to gain access to care.

**METHODS:**

Data was retrieved from the qualitative component of a multi-method research conducted with pregnant immigrants in Barranquilla, Colombia, between 2018 and 2019, and triangulated with a review of regulations established by the Ministry of Health and Social Protection.

**RESULTS:**

Having limited economic capital, participants use social capital from personal networks and migrant organizations. They obtain cultural health capital in the form of information on the health system and use their cultural competencies to interact with this system.

**CONCLUSIONS FOR PRACTICE:**

Migrants exert their agency through the use of capitals, although with certain constraints. Policies aimed at this social group should consider the strengths of migrants.

## INTRODUCTION

In recent years, over 4.3 million Venezuelans left their country^1^ in search for better opportunities or for escaping political instability. They are part of a mixed migrant flow, caused by a combination of economic, social, and political reasons, and are likely to remain in the receiving countries for relatively long periods.

Sharing a two thousand kilometers border with Venezuela, Colombia is the largest recipient of this flow. The number of Venezuelan immigrants in Colombia significantly increased after 2017, totaling more than 1.4 million in 2019 – 666,000 of whom were undocumented. The remaining migrants were regular, mainly holding a special residence permit issued by the Colombian government in response to the situation. National records also show that 48% of Venezuelan migrants were women, most of them of reproductive age^2^ , corroborating trends of feminization of international migration^3^ .

Healthcare in Colombia is fragmented, providing care according to employment status and income for different populations. As observed in other countries, financial limitations translate into infrastructure, personnel, and medication deficiencies that, combined with fragmentation, result in disparities in access and ineffective health coverage even for the national population^4 - 6^ . In response to the increasing Venezuelan mixed-migration flows, the Ministry of Health established that immigrants (including those in irregular situation) are entitled to emergency care and public health actions^7^ . Later on, access to prenatal care and other reproductive health services was also granted for undocumented migrants. In an attempt to provide healthcare access tantamount to that received by the Colombian population, Venezuelans holding a special residence permit can affiliate to an insurance program through their places of employment or be covered by the social assistance program for lower income or vulnerable populations.

Despite governmental actions, a recent article reported that 42.4% of undocumented pregnant Venezuelan migrants who had arrived to Colombia before the first trimester of pregnancy received no prenatal care. As for those who got pregnant in Colombia, only 22.1% received prenatal care during the first trimester^8^ . Another study shows that 76.8% of the 8,209 undocumented pregnant Venezuelan migrants registered in 2018 received no form of prenatal care^9^ .

Although international recommendations estipulate that migrants should have access to health services on equal terms with local populations^10 , 11^ , this is rarely the case, especially for irregular migrants, who have fewer resources to claim their rights, more enrollment constraints, and less knowledge of access routes as compared to regular migrants^12 - 15^ . The former are also more vulnerable to job insecurity and difficulties in accessing social protection systems^16^ , which translates into enhanced health risks.

Given the above, understanding the difficulties encountered by immigrants in accessing prenatal care in receiving countries and how they manage to navigate healthcare services is crucial. Although often explored in the context of migration from less developed regions to high-income countries, such issue has gained increasing prominence in the context of south-south mobilities, constituting a new panorama where people move out from their countries of origin due to factors associated with economic, political, violence-related, or climate change reasons to low- and middle-income countries with limited health systems. The migration of Venezuelans to Colombia illustrates one such case, providing an interesting example on how pregnant immigrant women fare in this context.

When studying migrant health, one must consider that migrants are not simply a vulnerable population, but that they are capable of mobilizing resources to seek for healthcare^17^ . In this scenario, studies must document how migrants exercise their capacity for action and the elements employed by them for navigating the health system without disregarding the barriers to access imposed by health systems and immigration regulations. To access services, people mobilize resources that constitute their economic and social capital^18 , 19^ and their cultural health capital^20^ . This paper uses these concepts as a theoretical framework for exploring how undocumented migrants use their capitals to access services, aiming to 1) explore the experience of irregular Venezuelan female migrants in search for access to prenatal care in Colombia; and 2) examine what resources they mobilize to gain access to services.

## METHODS

Data used in this study were retrieved from a multi-method project. Some quantitative results have been previously reported elsewhere^8^ . Our analysis is limited to the results of qualitative interviews conducted between 2018 and 2019 with 15 pregnant women in Barranquilla – one of the Colombian cities with the highest number of Venezuelan immigrants^2^ . Venezuelan, undocumented, pregnant migrants who were in Colombia for at least two months were included in the study through purposive sampling^21^ . Participants were located in public hospitals and areas with a high concentration of Venezuelans. Interviews were conducted in University offices for one hour on average, recorded, and transcribed for analysis. The following information were collected: living conditions, health problems, nutritional health and food safety, depressive symptoms, accessibility, quality and satisfaction with Colombian health services, and care-seeking trajectories. Table 1 shows participants’ characteristics.

**Table:** Participants’ characteristics.

Identification no.	Year of birth	Education level	Month/year of departure from Venezuela	Monthly household income^a^	Self-identification	Number of children
E01	1995	Middle school	03/2018	< 247 US	Mestiza	1
E02	1981	High school	06/2018	< 247 US	Mestiza	3
E03	1998	High school	01/2017	247–865 US	Mestiza	0
E04	1993	Tertiary	04/2018	< 247 US	Mestiza	1
E05	1996	High school	01/2016	247–865 US	Mestiza	0
E06	1989	Middle school	06/2018	< 247 US	Indigenous	2
E07	1992	Baccalaureate	12/2017	< 247 US	Mestiza	0
E08	1983	Post-secondary	12/2017	< 247 US	Mestiza	2
E09	1993	High school	09/2017	247–865 US	Mestiza	0
E10	1991	Tertiary	04/2018	247–865 US	White	0
E11	1998	Middle school	08/2017	247–865 US	Mestiza	1
E12	1999	Middle school	01/2018	247–865 US	Mestiza	1
E13	1988	High school	02/2018	< 247 US	White	0
E14	1995	Middle school	02/2019	< 247 US	Mestiza	2
E15	1997	Middle school	08/2018	< 247 US	White	0

^a^ Approximate value, considering an exchange rate of 3,356 Colombian pesos = one dollar.

For contextualizing interview results, policy documents issued by the Colombian Ministry of Health and Social Protection in response to the migration crisis were reviewed.

All procedures involving human subjects were reviewed and approved by the Institutional Review Board of the *Comité de Ética en Investigación* of the *División Ciencias de la Salud of the Universidad del Norte* (approval no. 170) to assure they were in accordance with ethical standards. All participants agreed to participate by an informed verbal consent, and the project covered for the expenses related to meal and transportation to the interview site (≈$6 US).

Transcribed data underwent a deductive content analysis – a research method for analysis and interpretation of qualitative material through the systematic process of coding and identifying patterns across cases^22 , 23^ – according to the following steps^24^ : early analysis for prior codes and categories; data coding; codes and categories review; comparison of emergent themes across cases; and conclusions. Using ATLAS.ti 8 and ATLAS.ti Cloud, three researchers adjusted the initial coding scheme, defined each code, and individually coded the interviews. Researchers held regular meetings to discuss the analysis process, revise and group the codes into categories, and identify emergent themes. After revising and coding all interviews, they discussed emergent themes and their relevance across cases and finally determined a final set of relevant aspects and conclusions that responded the research questions.

## RESULTS

### Limited Economic Capital and Material Resources: the Context of Migration and Pregnancy

Pregnancy and migration were interconnected in participants’ experience. Some individuals reported having migrated due to the difficulties of accessing basic products for medical and maternal healthcare in Venezuela, This finding is in line with the increase in maternal mortality in Venezuela due to the precariousness of maternal and child care^25^ .

Above all, I came for the medical attention (...) I was driven by both the pregnancy and my son’s disability... [IN VENEZUELA] when you go to give birth, you have to take your own gown, gloves, injection, and alcohol – everything they need to attend you, everything (...) It’s impossible to have a child in Venezuela. (E02)

We also found the economic and material resources of our study participants to be extremely limited (see Table 1), decreasing even further when pregnancy restricted their capacity to meet work exigencies. Some participants reported migrating due to economic reasons, as pregnancy directly impacted their working goals. According to some authors, this situation can increase both pregnancy- and migration-related stress^26^ . Inability to work and the consequent economic constraints (which in some cases led to food insecurity) recurrently appeared as a source of psychological distress in the narratives.

(...) I don’t go out, I don’t walk, I don’t do anything like that [NOW THAT I’M PREGNANT] because I know they won’t give me a job (...) When I don’t have anything (...), I go to a friend’s house and we eat there if I’m not embarrassed (...) if we have breakfast, we don’t have lunch; if we have lunch, we don’t have dinner (...) I: Are there days when you don’t eat? P: Sometimes I don’t eat anything. In other words, everything I get is for them [THE CHILDREN] (...), but we adults – their father and I – don’t eat. (E02)

Some participants expressed concern about living in houses considered unsuitable for a pregnant woman, mentioning inadequate infrastructure and basic services. The most frequently reported problem in terms of housing was overcrowding, which hampered women’s resting and constituted a source of stress due to conflicts among inhabitants.

I took a pregnancy test (...) and, well, I cried (...) I really didn’t want to have [THE CHILD] due to the situation in here. I mean (...) here we have to cook with wood, and the smell of charcoal gets everywhere in the house; (...) there is no water, no gas, because this house is sort of deemed as of high risk (...) Even though I was pregnant and didn’t feel well, I used to get up at four o’clock. Then I’d sit down for a while, make some tamales, sit down again and make some more tamales, and so on (...) My God, I don’t know how my baby will survive (...) with so much stress. I would like to be relaxed. (E08)

In a context of limited economic capital, public services constituted the main source of maternal healthcare. Below, we describe how access to healthcare depended on the changes introduced by the Ministry of Health and Social Protection in response to Venezuelan immigration, and how participants mobilized their capitals for navigating the system.

#### Navigating a changing health system: social and cultural health capital

In Colombia, only regular immigrants are entitled to enroll in the General System for Health Social Security (SGSSS) insurance scheme. In 2018, the Ministry of Health and Social Protection granted the right for undocumented immigrants to receive emergency care and participate in public health actions such as prenatal check-ups and childbirth and postnatal care^7^ . Venezuelans migrants had to obtain a residence permit and affiliate to the SGSSS to obtain access to maternal healthcare, or follow the special administrative procedures at the local level to comply with the Ministry’s resolution.

During the period of this research, these administrative procedures underwent numerous changes. In Barranquilla, pregnant women had to bring a pregnancy test result and their identity document to the City Hall to receive authorization for accessing prenatal care at the local public hospital. For each medical procedure, a new authorization had to be requested, so that women could be required to go to the City Hall multiple times during pregnancy. At first, only 25 authorizations were issued daily, and some participants reported spending the night at the place and leaving without an authorization. In response to this problem, the government created a telephone line for appointments, but quotas remained insufficient, so that many respondents were unable to access prenatal check-ups timely.

The first time, I spent two days ringing continuously and they only answered at about half past three in the afternoon of the second day – after I had called about 40 times. It was not as complicated at the second time – I got through on about my fifth try. However, this time I have been calling every day and they have not answered me for a month even though I have called from Monday to Friday. (E04)

Many participants reported that the procedures for regularizing their documents and affiliating to the SGSSS were likewise unclear and burdensome. As a result, some migrants gave up on the process and relied on emergency services as source of medical attention. Most migrants using the country emergency services reported very few obstacles; however, others were refused care on the basis of their status as migrants, which may have been due to the negligence or ignorance of health professionals regarding the new provisions.

Participants coped with all these barriers by looking for strategies to access services, including the mobilization of social capital, both from personal support networks (partner, family or friends) and community organizations. With these resources, participants managed to access medical care or medications more easily; as stated by one of them: “Here you see what you can do on a day-by-day basis” (E02). The collective influence of migrant organizations not only influence authority decisions, but also support their female participants in overcoming problems related to healthcare access.

(…) Due to a pregnant women meeting that we organized, word spread and the undersecretary of health became interested in the foundation [MIGRANTS’ ORGANIZATION], went to a meeting, and saw our work. She also mentioned it to some colleagues such as the registrar and told them it was a serious foundation that had existed for some time and already worked with several government agencies. Then, she talked to us and said she could help us a bit and get these girls admitted more quickly than in the usual system (...) (E03)

To access healthcare, participants also leveraged their cultural health capital, which, according to Shim^20^ , includes information on the health system and incorporated knowledge on how to interact with health workers and other bureaucrats. As for information, given the changes in the administrative procedures and regulations concerning access, respondents required up to date information. To this end, they resorted to both formal and informal sources, including friends, family and neighbors, other pregnant Venezuelans, social networks, meetings in the waiting rooms of hospitals and offices, and health service workers. Among the latter, those with knowledge of the access routes to services became especially important.

(…) Then, I asked her if there was a social worker, because they always tend to speed up the process or tell us what to do. (E03)

Because another Venezuelan at the hospital explained it to me; that is, the girl, the woman who told me about the ultrasound scan, told me “when you get your ultrasound done, you have to go to City Hall”. There was a girl who had just given birth there who said, “That’s not true. You have to call to make an appointment.” I was told by another Venezuelan woman who was there. (E04)

As for knowledge on how to interact with the system, given that bureaucrats at various levels have a certain degree of discretion in their functions^27^ , participants used their interaction skills to negotiate their position by being either friendly or polite to the officials or by emphatically demanding attention – the former attitude being more common than the latter.

I: What kind of treatment do you get? P: A good one. I mean, they were attentive. The point is: *you also have to know how to treat people,* because some people say [IMITATING RUDE TONE] “Look how far this place is [FROM PARTICIPANT’S HOUSE]”. So, *if you don’t treat people politely, you won’t be treated politely.* (E04) [OUR ITALICS]

As part of their knowledge of interaction modes, the interviewees knew that getting attention from health workers outside the formal channels was yet another option for receiving access:

... they told me they couldn’t see me there because I didn’t have an insurance card (…) they couldn’t help me. But a nurse overheard me and told me to talk to the doctor privately, so I did. I talked to the doctor privately and explained my situation, so she treated me, she treated me there. I: So, she gave you a sort of informal consultation. P: Exactly, yes. And she told me (…) well, she gave me some recommendations, she prescribed some injections, I bought them, and she gave me the injections (…) but it wasn’t a proper consultation (...) (E02)

In short, participants managed to access the required prenatal care by mobilizing capitals. However, they still experienced several difficulties in accessing healthcare and most times it was not timely.

## DISCUSSION

In analyzing how migrants with limited economic and material resources managed to access health services, we found that their access was based mainly in social capital and cultural health capital. Social capital, defined as social relations based on trust, reciprocity, and cooperation^19^ , enabled them to navigate the health system thanks to networks inside and outside the migrant community. In turn, cultural health capital helped them to discover access routes and interact with providers. In this sense, our results highlight the agency of migrant women^17^ and the possible effects of this agency on the health system^28^ .

However, the importance of these capitals for healthcare access reflects the limitations in care. Integration policies (including access to healthcare) play an essential role in guaranteeing the rights and health of migrants^29^ , and the limitations inherent to these policies indicate structural problems in the health system. Despite the efforts exerted by the Colombian state to guarantee effective access to care^4^ , the health system has limitations regarding timeliness and quality^6^ , and the complexity of bureaucratic procedures hamper access to care^28^ , especially regarding undocumented migrants^30^ . Our results show that, even after being legally recognized as a right, the access to prenatal care is still limited due to bureaucratic procedures and insufficient information.

Migration and health system characteristics are social determinants^31 , 32^ that either limit or increase decision-making and capacity for action among women. In our study, migrants operated within a restricted framework of possibilities, requiring greater investments of resources for accessing services when compared to less vulnerable people. Such a need for investing a large amount and diversity of resources for meeting basic needs is a constant in the subsistence of vulnerable social groups^33^ and comprises a social issue that should be solved within the framework of universal health coverage.

Our study has some limitations. First, as our sample comprises a specific group of pregnant women within a limited geographical site and considering that the experiences of regular Venezuelan migrants, in other contexts, or with better socioeconomic conditions may be different, our results cannot by generalized. However, participants’ narratives achieved theoretical saturation with a relatively low number of interviews, corroborating the results reported in other studies on undocumented migration and access to services^16 , 17 , 26^ , suggesting that the results obtained in our study would apply to other groups of migrants. Further research should explore these issues in other south-south migration contexts and include more diverse samples to improve generalizability.

Although migrants can respond in creative ways to obtain access to healthcare, authorities must keep promoting enrolment in insurance schemes on equal terms with the local population to guarantee these populations’ right to health. As in Colombia the main route for ensuring this equality is through regularization, governmental authorities should adopt a migration policy that facilitates such process, thus expediting access to health services. Moreover, information on health services and the necessary steps for accessing them should be disseminated among both migrant communities and service providers. Actions aimed at improving access to maternal health services for migrant women (while considering these group strengths) can be used to review and improve the health system, benefiting the entire population.
